# Longitudinal mixed methods study assessing caregivers of seniors across diverse populations: research protocol

**DOI:** 10.1186/s12913-020-05244-z

**Published:** 2020-05-01

**Authors:** Afifa Mahboob, Erin Relyea, Jill I. Cameron, Lisa Manuel, Alex St. John, Maria Huijbregts

**Affiliations:** 1 Family Service Toronto, 355 Church St, Toronto, ON M5B 0B2 Canada; 2 University of Toronto, 160-500 University Ave, Toronto, ON M5G 1V7 Canada

**Keywords:** Caregiver, Senior, Diverse communities, Mixed-methods, Workshops, Needs assessment

## Abstract

**Background:**

Canada’s aging population is increasing, along with the number of caregivers providing support to seniors. Caregiving is a taxing responsibility that often results in loneliness and distress. Creating awareness of available supports for caregivers is essential for their health and to provide the best support to the care recipients. This study aims to better understand and improve the caregiving experience for caregivers from diverse ethnic communities and the LGBTQI2S+ communities. The goal is to improve the well-being and resilience of caregivers and optimize outcomes for care recipients by delivering educational workshops that resemble the design of existing workshops currently offered by the participating social service agency. Content will be adapted based on identified participant learning needs. These workshops will be offered to the English-speaking community, diverse newcomer ethnic groups and the LGBTQI2S+ community.

**Methods:**

This mixed-methods, longitudinal study includes two streams of caregivers; Stream One consists of English-speaking caregivers and care recipients while Stream Two includes individuals from the Afghan, Iranian, Somali-, Tamil- and Spanish-speaking populations and those belonging to LGBTQI2S+ communities. Each stream has two phases; Phase One includes needs assessments using focus groups and semi-structured interviews with caregivers and care recipients while Phase Two includes a pre-test post-test evaluation of educational workshops. The anticipated sample size for Phase One is 30 caregivers from the English-speaking community, 150 from the five linguistic/cultural communities combined and 30 from the LGBTQI2S+ group. For Phase Two, we plan to recruit 250 caregivers from the English-speaking community, 250 from the five linguistic/cultural communities, and 50 from the LGBTQI2S+ group.

**Discussion:**

To provide caregivers with optimal support, we must acknowledge the caregivers and care recipients from diverse communities. Currently, at least two focus groups have been conducted with caregivers from each of the seven targeted groups and workshops have begun for all communities. Recruitment has been a challenge for all groups, but our team continues to conduct outreach with caregivers and will use our learning to inform the delivery of educational caregiver workshops.

## Background

With Canada’s population aging, the number of caregivers supporting seniors is simultaneously increasing. It is estimated that 3.8 million caregivers in Canada assist seniors with short or long-term health issues and approximately half a million are caregivers for a senior with dementia [1]. One report states that 10% of caregivers of seniors in Canada provide more than 30 h of support a week, 60% of whom are employed and 25% are also caring for a child [1]. In 2012 approximately 8 million Canadians, or 28% of people over the age of fifteen, provided caregiving to someone living with a chronic health condition [2]. Women are more likely than men to provide care, especially when caregivers are under the age of 65 [3]. While support may be available, many caregivers find it challenging to access the appropriate services that best suit their needs [1]. Caregiving is often a lonely and isolating task resulting in distress, anger or depression. Left unaddressed, this can lead to caregivers feeling overwhelmed and unable to continue in their role [4].

A large social service agency in Toronto has been providing caregiver education, training and counselling support to informal and formal (e.g. paid) caregivers for over 25 years. The population largely using this service are English-speaking caregivers, possibly creating difficulties for caregivers from different linguistic or cultural communities to address their needs. In addition, caregivers and seniors identifying with the LGBTQI2S+ community may not be aware of this service. Over this time, we have learned that the nature of caregiving differs from community to community and from person to person.

This study was carried out by the abovementioned social service agency. We will refer to this social service agency as ‘the agency’ in this article. The agency is offering a range of services for seniors and their caregivers from diverse populations in Toronto. The agency worked closely with a long-term care home in a lower income neighbourhood in Toronto to facilitate workshops for the program. The study will address the gaps in services provided to diverse caregivers by identifying caregiving needs from various communities and recommending services that are adapted to their specific concerns. For the purposes of this study, we developed two streams of caregivers to support different communities.

### Aim of study

The study aims to improve the caregiving experience for caregivers from diverse ethnic communities and the LGBTQI2S+ communities. Ultimately, the study is expected to enhance the well-being and resilience of caregivers and optimize outcomes for care recipients by identifying caregiver learning needs and delivering educational workshops to caregivers from all participating communities.

## Methods

### Study design

This mixed-methods, longitudinal study uses both qualitative and quantitative methods. The project consists of two streams of participants, each of which have two phases; the first one involves a thorough needs assessments and the second one will evaluate education, training, and support. The needs assessment is qualitative and uses descriptive methods such as focus groups and semi-structured interviews [5]. The quantitative portion uses a pre and post-test design comprising of validated outcome assessment tools used for Stream One participants as well as workshop feedback forms to assess the effectiveness of the intervention phase (phase two) of the study. For both phases, demographic information will be collected. Each stream has two phases, which consist of different activities and data collection tools. Table 1 describes the two streams of participants, phases and overall study design.

**Table 1:** Study Design

Streams	Phases	Activity	Information Collected
Stream 1 – Caregivers and care recipients from English-speaking community	Phase 1	- Needs assessment with caregivers in the form of focus groups - Semi-structured interviews with care recipients	- Demographic information - Qualitative focus group and interview feedback
	Phase 2	- Educational workshops for caregivers	- Demographic information - Workshop feedback forms - Outcome assessment tools (T1, T2, T3)
Stream 2 - Caregivers and care recipients from Afghan, Iranian, Somali-speaking, Tamil-speaking, Spanish-speaking community groups and the LGBTQI2S+ community	Phase 1	- Needs assessment with caregivers in the form of focus groups - Semi-structured interviews with care recipients	- Demographic information - Qualitative focus group and interview feedback
	Phase 2	- Educational workshops for caregivers	- Demographic information - Workshop feedback forms

### Intervention

Workshops will be created with the community facilitators from each of the communities using the focus group outcomes. Currently, the team has outlined five general themes from the data: what is the aging process, what is caregiving, communication between the caregiver and care recipient, where to find resources and caregiver self-care. Each community facilitator will use these themes as a starting point to develop their workshop content. The community facilitators will use culturally nuanced material to create and facilitate their workshops in a learning style that is preferred by their community. For example, the Iranian community favours a lecture style followed by discussion time, whereas the Spanish-speaking community prefers sharing their experiences using a talking piece, which is passed around to each participant to help facilitate the conversation. Resources and content will be sourced and shared between community facilitators when available; however, the main goal is to ensure that linguistic and community needs are met.

### Participants

As identified in Table 1**,** Stream One consists of English-speaking caregivers and care recipients while Stream Two includes caregivers and care recipients from the Afghan, Iranian, Somali-speaking, Tamil-speaking and Spanish-speaking populations as well as individuals belonging to the LGBTQI2S+ communities.

#### Inclusion criteria

Caregivers include individuals who are caring for a senior, aged 55+. Caregivers must be 18 years or older. Caregivers attending English-speaking focus groups and workshops are expected to understand English. Caregivers from the diverse linguistic/cultural communities must identify with those communities and speak and understand the common language. For focus groups and workshops with the LGBTQI2S+ population, all participating caregivers must be caring for a senior (55+). At least one focus group must be specific to the transgender community and one focus group with heterosexual caregivers who provide formal or informal care to LGBTQI2S+ seniors. Care recipients recruited for semi-structured interviews must belong to the LGBTQI2S+ group and be ages 55 + .

#### Exclusion criteria

Care recipients below the age of 55. Caregivers or care recipients who have a condition that may limit their ability to communicate, including stroke, aphasia, dementia, or more. These conditions will be determined by the facilitator on an individual basis.

#### Sample size

The sample size for the focus groups is approximately 30 caregivers from each participant group (the English-speaking community, the five linguistic/cultural communities, and LGBTQI2S+ community) for a total of 210. This sample will allow theme saturation within each community to facilitate exploration of differences between groups. Three to five care recipients from each community will also be selected for semi-structured individual interviews to provide any additional insight.

The anticipated sample size for the workshop evaluations is approximately 126 caregivers from the English-speaking community. The CESD-10 was the main outcome tool [6]. Using a paired t-test and expecting a change of 2.0 (SD of 6.5), we would need to have 126 participants in order to have 80% power with a two-tailed alpha (.05) [7, 8]. Caregivers from each of the five linguistic/cultural communities, and caregivers from the LGBTQI2S+ group only completed workshop feedback forms.

### Recruitment

#### Phase one

A purposive, convenience sample of caregivers and seniors from the identified communities will be recruited to participate in focus groups and semi-structured interviews. The study seeks participants from the diverse communities identified, which is why a purposive sampling method was used. Participants will be recruited from a list of current and past clients of the aforementioned agency and from the community at large. Recruitment includes the following strategies and sources:

- Hospitals, community programs, community health centres, family health teams etc.
- Social media feeds
- Email blasts
- Flyers
- Posters
- Agency website and programs
- Ethno-specific radio broadcasts
- Faith communities
- Word of mouth

#### Phase two

Participants will be recruited from a list of current and past clients of the agency and with the use of similar recruitment strategies and sources, as mentioned in Phase 1.

Participants from Stream One who are interested in attending at least three workshops will be invited to complete validated outcome assessment tools.

### Data collection

All participants will provide informed consent that will be explained verbally and in written format. All participants are expected to provide demographic information. All data collection and caregiver workshops will be conducted in a private room and led by the Project Coordinator or the specific community-based facilitators.

Data will be collected using both qualitative and quantitative methods. Data sources include focus groups, semi-structured interviews, workshop feedback forms and outcome assessment tools. The data collected from focus groups and interviews from Phase One of the study will be used to generate topics for the workshops in Phase two. The use of the tools varies depending on the stream of caregivers.

#### Stream one

After the focus groups with English-speaking caregivers and interviews with care recipients in Phase One of the study, Phase Two of the project will be launched. This consists of workshops with caregivers. At the end of each workshop, workshop feedback forms will be distributed to better understand the caregiver experience and evaluate the impact of the workshops. Workshops will be offered once a month at the three agency locations or in community settings outside these locations, as time and logistics permit. In addition, a partnership has been developed with a long-term care home, which will provide workshops on various physical aspects of caring (e.g. medication management, mobility, etc.).

All focus groups, interviews and workshops will be conducted in a private room and led by the Project Coordinator. Informed consent and confidentiality will be explained both verbally and in writing. Demographic information is also collected from caregivers and care recipients during all phases.

#### Stream two

Stream Two of the project consists of conducting focus groups and semi-structured interviews with caregivers and care recipients within the diverse communities and the LGBTQI2S+ community. Each community will conduct at least three focus groups with caregivers and three semi-structured interviews with care recipients. Care recipients will be interviewed because the study requires a small sample of care recipients as the primary focus is to hear from more caregivers from each community. In addition, interviews give the option to connect with care recipients in their own homes or an easily accessible location for them, as some suffer from various health conditions or do not have the ability to travel far for focus groups. These focus groups and interviews will be spread out over time to help develop workshop topics and to determine the impact of the workshops and overall study.

The English versions of the demographic form, informed consent form and workshop feedback form will be translated by each community facilitator and distributed to all participants in the same manner as explained in Stream One.

#### Outcome assessment

In order to test the impact of the workshops for caregivers and their well-being, validated outcome assessment tools that measure various caregiver abilities will be used. The outcome assessment tools consist of a collection of scales: depression scale (CESD-10) [6], caregiver assistance and confidence scales [9], caregiver impact scale [10], personal gains scale [11], mastery scale [12] and an adapted health resource utilization scale [13, 14]. The tools will only be administered to the English-speaking community (Stream One participants) during Phase Two of the project as translated versions of the tools are not available.

The goal is to administer the tools three times (T1, T2, T3) to assess outcomes before attending workshops (pre-assessment), during attendance of any third workshop, and finally after attending the last workshop (post-assessment) (refer to Table 1**).**

#### Final focus group with facilitators

After all phases of the project are complete, a final focus group will be held with the six community facilitators and project coordinator to learn about their experiences, their suggestions for next steps and their overall feedback on supporting caregivers in Toronto. The focus group will be facilitated by staff from the agency’s research team. This includes one facilitator and one note-taker. This focus group will help summarize the qualitative data and create ongoing discussion on how to better support caregivers of seniors.

### Data analysis

#### Analysis of quantitative data

Quantitative data collected from workshop feedback forms and the outcome assessment tools will be uploaded to password-protected computers for analysis by the research team. The statistical analysis will include descriptive analysis, group comparisons, analysis of variance and T-tests. Analyses will include variations based on ethnicity, sexual identity, age, sex and other significant variables that may be associated with caregiver needs and experience.

#### Analysis of qualitative data

All notes produced from focus groups and semi-structured interviews will be analyzed by the research team. A thematic approach will be used to identify caregiver needs and to further improve services provided to them [15]. This will allow the research team to compare the needs of caregivers from different communities. Needs include challenges, barriers and gaps in caregiver services. Comparisons will be done by identifying common themes suggested by caregivers and care recipients from the different communities. In addition, some communities may have needs and concerns that are specific to their communities, which will also be included in final reporting. A table will be created that will identify the themes, along with a corresponding description. Data will include direct quotes from caregivers and care recipients, as well as paraphrased concepts. This approach will allow us to adapt programming to specific community-based needs.

## Discussion

Caregivers require appropriate resources to help them care for themselves and their care recipients. To provide them with optimal support and to best address their needs, it is important to acknowledge the diverse communities that caregivers and their care recipients belong to. By targeting caregivers from various communities, this project seeks to address the needs of caregivers from different linguistic groups and those belonging to the LGBTQI2S+ community.

For both the focus groups and workshops, recruitment has been a challenge, particularly for the English-speaking group. Caregivers have displayed a lack of interest in completing the outcome assessment tools because they found the tools to be too lengthy. While workshop attendance for the English-speaking community is gradually increasing, very few caregivers have completed the outcome assessment tools. This greatly limits the quantitative evaluation of the study; however, the richness of the qualitative learning will be a contribution to the body of literature in this area.

The other community groups are struggling to find participants who are comfortable completing all the required documentation for the study, including demographic forms and consent forms. Participants have expressed that the forms are lengthy or are uncomfortable answering certain demographic questions. Since the approved study protocol expects these forms to be completed, some potential participants have refused to participate. This has affected our ability to recruit for the study, even when people attend the program.

Despite these challenges, our team continues to do outreach to recruit caregivers and will reassure them about the confidentiality and anonymity of their participation in the study, the value of providing their feedback during focus groups and the educational benefits of participating in workshops.

## Data Availability

The dataset supporting the conclusions of this article is not publicly available because the participants involved in this study are clients of the social service agency; clients belong to vulnerable populations and have been guaranteed that their information will be protected and their feedback will only be provided in an anonymous, aggregated and summarized format. Data can be made available from the corresponding author and with permission from the social service agency upon reasonable request, but participant information will remain anonymous.

## Abbreviation

LGBTQI2S+: Lesbians, Gay, Bisexual, Transgender, Queer/Questioning, Intersex, Two-Spirit and others
